# Italian version of Nursing Students’ Perception of Instructor Caring (I-NSPIC): assessment of reliability and validity

**DOI:** 10.1186/s12909-017-1032-y

**Published:** 2017-11-17

**Authors:** C. Arrigoni, M. Puci, A. M. Grugnetti, L. Collivasone, E. Fenizia, P. Borrelli, E. Vellone, R. Alvaro, M. Piredda, M. G. De Marinis

**Affiliations:** 1 Department of Public Health, Experimental and Forensic Medicine, Unit of Hygiene, University of Pavia, Via Forlanini, 2-27100 Pavia, Italy; 2 Department of Public Health, Experimental and Forensic Medicine, Unit of Biostatistics and Clinical Epidemiology, University of Pavia, Via Forlanini, 2-27100 Pavia, Italy; 3 IRCCS Policlinic San Matteo Foundation Pavia, Via Forlanini, 2-27100 Pavia, Italy; 4 Azienda Ospedaliera di Pavia, Corso Milano, 19, Vigevano, Pavia, Italy; 5 Tor Vergata University, Faculty of Medicine, Via Montpellier, 1, 00133 Rome, Italy; 6 Research Unit Nursing Science, Campus Bio-Medico di Roma University, Via Alvaro del Portillo, 21-00128 Rome, Italy

**Keywords:** Caring, Instructor caring, Nursing education, Clinical placement, Nursing students’ perception

## Abstract

**Background:**

Clinical experience is an essential component of nursing education since it provides students with the opportunity to construct and develop clinical competencies. Instructor caring is a pivotal facilitator at the forefront of clinical education, playing a key and complex educating role in clinical sectors. For these reasons the aims of this study was to assess the validity and reliability of the Italian version of NSPIC (I-NSPIC).

**Methods:**

A validation multicentre study was conducted in three different Italian universities. A total of 333 nursing students were enrolled in the 2014/2015 academic year. Exploratory factor analysis (EFA) with oblique rotation was performed to test the construct validity of I-NSPIC. The Cronbach’s alpha coefficient and test retest via Intraclass Correlation Coefficient (ICC) analyses were done to assess the internal consistency and stability of the scale. A Spearman’s correlation with another scale (CLES-T) was used to examine the concurrent validities.

**Results:**

Four factors (control versus flexibility, supportive learning climate, confidence through caring, appreciation of life meaning and respectful sharing) were identified in EFA. The Cronbach’s alpha value showed that I-NSPIC was a reliable instrument (α = 0.94) and the ICC coefficient was satisfactory.

**Conclusion:**

The I-NSPIC is a valid instrument for assessing the perception of instructor caring in Italian nursing students. It may also prove helpful in promoting the caring ability of nursing students and in increasing the caring interactions in the relationship between instructor and nursing students.

The knowledge emerged from this study provide important insight in developing effective training strategies in the clinical training of undergraduate nursing students.

**Electronic supplementary material:**

The online version of this article (doi:10.1186/s12909-017-1032-y) contains supplementary material, which is available to authorized users.

## Background

In nursing education clinical learning and competency development are essential parts of the nursing curriculum. Clinical experience is an essential component of nursing education since it provides students with the opportunity to construct and develop clinical competencies and apply assistance to a real-world context, thereby allowing students to transfer theory into practice [1–7]. In recent years, caring is gaining scientific dignity and operational relevance and is believed to be a core component of nursing education [8–14].

Jean-Watson’s studies in interpersonal care emphasize that patients experience greater emotional and spiritual well-being, improved healing, and greater confidence in their relationship with health professionals when they experience a careful and participative care relationship [11].

The philosophy of the humanist paradigm is put into effect in the constructs of care, learning, participation and reflection. The movement toward this paradigm requires a transformation to a more egalitarian student-teacher relationship, thereby supporting the development of both partners, in order to understand students and facilitate their learning [15]. Therefore, instructor caring is a pivotal facilitator of clinical education [16, 17], serving as a model for the student [18, 19] and playing a key and complex educating role in clinical sectors involving many personal, interpersonal and organizational aspects [20, 21]. Moreover, instructors’ caring facilitates the transition from the students’ role to the nurse role [22, 23], where the instructor works with students on their learning experiences as a coach and mentor [15].

Students award a fundamental value on the clinical environment in terms of their professional development, and their instructor caring relationship, important elements for the success of their internship [16, 20, 24–26].

Emphasis on caring theories should be placed in nursing education and their application in nursing practice. Watson’s theoretical framework, which focuses on interpersonal and transpersonal processes in human care, presents an effective model in understanding the concept of caring.

Watson argued that caring is the main concept in nursing education. The aim of the caring based curriculum is to provide students an overall view of caring, so that they will be able to care for the health of the individual as nurses [8, 11, 13].

Wade and Kasper [27], feel that nursing education is an ideal place to promote and develop student caring because this is where personal involvement with others occurs.

Wade & Kasper [27] argue that the NSPIC, based on Watson’s Theory of Transpersonal Caring, is an extremely valid and reliable tool to evaluate students’ perception of instructor caring. Moreover, they believe this tool can be used as an outcome of the educational process to validate the influence of support interactions between students and instructors caring about the students’ caring capabilities. Caring interactions between teachers and students reflect the same nature of the nurse-patient relationship [15, 28]; therefore, nursing students’ perceptions of instructor caring can reveal how nursing students learn to care for their patients. If students perceive an educational environment based on care, they will learn to become professionals [8, 25, 27].

Caring clinical environment improves well-being in nursing students, self-confidence, motivation and decreases anxiety associated with the clinical setting, facilitating the learning [8, 29–31]. Providing for a high-quality clinical environment is a determining factor in improving the quality of health services [31, 32].

The Nursing Students’ Perception of Instructor Caring (NSPIC) instrument, based on Watson’s Theory of Transpersonal Caring, was developed to evaluate nursing students’ perception of instructor caring in the clinical setting in the U.S.A. [27] and subsequently validated in China [33] and Iran [34]; it was used in Italy but not validated. However, in Italy, currently no studies exist investigating the impact of instructor caring on nursing students; therefore, in this study we are interested to validate the NSPIC in Italy for assessing the perception of instructor caring among Italian nursing students. The use of this tool could help to identify the critical issues that need work to improve the quality of the nursing students’ clinical learning.

The aims of this study were to translate NSPIC into Italian and to examine the reliability and validity of Italian version of NSPIC (I-NSPIC) in nursing students during clinical practice, to obtain a tool that allows us to evaluate students’ perceptions of their tutor to improve the quality of clinical training in the Italian context.

## Methods

### Study design, participants and setting

We conducted a validation multicentre study. A sample of 333 junior (I and II years) and senior (III year) nursing students in bachelor’s degree nursing programs, enrolled in the academic year 2014/2015, was randomly selected from three different Italian universities: Pavia, Roma Campus Biomedico and Roma Tor Vergata. Researchers who administered I-NSPIC were not the same as those that evaluated students in clinical laboratories. Students completed a questionnaire in a university classroom.

### Description of the original instrument

NSPIC is an English questionnaire composed of 31 items divided into five dimensions: instills confidence through caring, supportive learning climate, appreciation of life’s meanings, control vs flexibility, and respectful sharing. Each item is assessed on a 6-point Likert scale (responses ranged from 1 “strongly disagree” to 6 “strongly agree”). To explore the structure of the questionnaire an Exploratory Factor Analysis (EFA) was utilized; five factors were identified which explained 71.7% of the variability. NSPIC and its subscales showed very good internal consistency (NSPIC’S Cronbach alpha = 0.97, factor 1 α = 0.96, factor 2 α = 0.94, factor 3 α = 0.89, factor 4 α = 0.72 e factor 5 α = 0.75). The total NSPIC score ranges from 31 to 186, with higher scores representing greater perceptions of instructor caring.

### The screening scale translation process

A forward-backward translation procedure was applied to establish content validity of the NSPIC scale. Firstly, the NSPIC was translated into Italian and submitted to an expert panel (four expert nurses in nursing education and research) that compared the original English version with the Italian one to ensure the semantic and cultural coherence of the items. Secondly, an English lecturer translated the Italian version back into English as a blind. Finally, the back-translate instrument and the original one were compared by the NSPIC’s authors to establish the content validity of the double translated instrument (Additional file 1). The authors agreed that the Italian version was identical in meaning to the English version [35].

### Ethical considerations

We obtained permission to validate the Italian version of the NSPIC from the authors of the original questionnaire, whom we contacted by e-mail. This study was given written approval by the University Academic Committee. All of the participants were given detailed information regarding the study aims and methodologies. The questionnaire used in this study was anonymous, and verbal consensus was obtained before its compilation.

### Statistical analysis

To identify the required sample size we applied the n:p rule of thumb [36]; the n:p is the minimum recommended ratio of sample size (n) to number of variables being analysed (p). Everitt [37] recommended that the n:p ratio should be at least 10. In the present study, in which *n* = 333 and *p* = 31, it was 10.74, so our final sample size was in the recommended value.

Quantitative variables were summarized as mean values, standard deviations, median and interquartile range (Iqr), and qualitative variables as percentage frequencies. We assessed the factorial structure of the questionnaire by EFA, a multivariate statistical technique [38]. To determine the number of factors to retain we applied Kaiser – Guttman “Eigenvalues greater than one” criterion [39–41] and Cattell’s scree plot [42]. Oblique rotation (*promax rotation*) was used in the EFA because the factors were expected to correlate [43]. Factor loadings ≥0.4 were considered as significant and were entered in the final questionnaires [44]. In order to test unidimensionality of the subscales we again ran a factor analysis. We created a score for the I-NSPIC questionnaire (sum of the responses of 31 items) and for its subscales (sum of the responses related to each dimension). Normality distribution was assessed using the Shapiro-Wilk test. To compare scores between groups we used a parametric Student T test for independent data or corresponding non-parametric test (Mann-Whitney or Kruskal-Wallis test); for all the analyses we considered as a significant level *p* < 0.05. All analyses were conducted using STATA/SE for Windows, version 12.1 (StataCorp, college Station, TX, U.S.A.).

### Validity and reliability

The validity of a questionnaire represents the degree to which it measures what it is meant to measure. There are different types of validity; criterion-related validation investigates possible associations between the examined scale and external criteria or other validated measures. Therefore, we correlate the I-NSPIC score using Spearman’s correlation coefficient with the Italian version of “*Clinical Learning Environment and Supervision plus nurse Teacher scale”* (CLES-T) and its subscale “*Supervisory relationship”* (related to the content of the supervisory relationship between tutor and student) [5], which represents a gold standard Additional file 2. Prior to data collection, permission to use the CLES-T was granted by its authors through email.

Internal consistency was determined by calculating Cronbach’s alpha coefficient [45]. Test-retest reliability between two subsequent administrations of the questionnaire, separated by an interval of 30 days, was assessed in a subset of the sample (25 students) using an intraclass correlation coefficient (ICC) [46].

## Results

NSPIC scale and back-translation process were successful.

The demographic characteristics of the sample are summarized in Table 1.

**Table 1:** Sample characteristics

Variables	N (%) 333 (100)
Age (mean, standard deviation)	22.66 ± 4.16
Sex	
Female	269 (81)
Male	64 (19)
Nationality	
Italian	321 (96)
Foreign	12 (4)
University	
Pavia	127 (36)
Rome – Campus Biomedico	120 (38)
Rome – Tor Vergata	86 (26)
Education	
Junior (I–II years)	228 (68)
Senior (III year)	105 (32)

### Factorial structure

The number of eigenvalues >1 computed by EFA totaled four. The percentage of cumulative variability explained by a model with four dimensions is 62.92%: Factor 1 explains 42.5% of variability with an eigenvalue of 13.17 (Table 2). The subscales were unidimensional; therefore, each subscale measured only one construct. In addition, Table 2 shows factor loadings for each item.

**Table 2:** Exploratory factor analysis results for NSPIC (*N* = 333)

	Factor 1	Factor 2	Factor 3	Factor 4
Eigenvalue	13.17	3.96	1.21	1.16
% of variability	42.50	12.78	3.90	3.74
% of cumulative variability	42.50	55.28	59.19	62.92
Item				
Supportive learning climate				
3. Instills in me a sense of hopefulness for the future.	0.5397	0.4020		
8. Cares about me as a person.	0.7399			
13. Acknowledges his or her own limitations or mistakes.	0.4991	0.3614		
15. Clearly communicates his or her expectations.	0.5505			
16. Serves as a trusted resource for personal problem solving.	0.9149			
17. Offers support during stressful times.	0.8877			
18. Accepts my negative feelings, while helping me to see the positive aspects.	0.8737			
19. Allows me to express my true feelings.	0.8278			
21. Inspires me to continue my knowledge and skills development.	0.4810	0.4749		
27. Helps me find personal meaning in my experiences.	0.7985			
28. Encourages me to see others’ perspectives about life.	0.7643			
29. Helps me understand the spiritual dimensions of life.	0.8433			
Instills confidence through caring				
1. Shows genuine interest in patients and their care.	0.3756	0.4895		
2. Displays kindness to me and others.	0.3469	0.5330		
4. Makes me feel that I can be successful.	0.3982	0.5281		
5. Helps me envision myself as a professional nurse.	0.4253	0.4753		
9. Respects me as a unique individual.	0.4179	0.4896		
10. Is attentive to me when we communicate.		0.5048		
14. Makes himself or herself available to me.	0.4240	0.5166		
Respectful sharing				
6. Makes me feel like a failure.			0.9135	
7. Does not believe in me.			0.8143	
12. Does not reveal any of his or her personal side.		0.4621	0.4796	
20. Discourages independent problem solving.			0.5076	
22. Makes me nervous in the clinical laboratory.			0.7323	
23. Does not trust my judgment in the clinical laboratory.			0.6130	
24. Seems caught up in his or her own priorities, rather than responding to my needs.			0.4581	0.3261
Control vs flexibility				
11. Inappropriately discloses personal information about me to others.				0.3230
25. Makes demands on my time that interfere with my basic personal needs.				0.6160
26. Focuses on completion of patient care tasks, rather than the patient’s needs.				0.5402
30. Is inflexible when faced with unexpected situations (happenings).				0.8341
31. Uses grades to maintain control of students.				0.4884

### Validity

In references to criterion-validation, Table 3 shows the Spearman’s correlation coefficients between the score of I-NSPIC and its subscales and the CLES-T questionnaire and its subscale “Supervisory relationship”. All the coefficients were significantly different from 0 (*p* < 0.05) (Table 3).

**Table 3:** Spearman correlation coefficients between the scores of the NSPIC and its subscales and between the CLES-T gold standard questionnaire and its subscale Supervisory Relationship

	CLES-T	Supervisory Relationship (CLES –T subscale)
NSPIC (sum of 31 items)	0.54	0.50
Supportive learning climate	0.67	0.66
Instills confidence through caring	0.59	0.65
Respectful sharing	−0.38	−0.40
Control vs flexibility	−0.25	−0.32

The mean score for I-NSPIC is 113.63 (sd 19.09, range 31–186). Table 4 shows mean score, standard deviation, median and Iqr of I-NSPIC by sex, university and education.

**Table 4:** Mean, sd, median and Iqr of NSPIC by sex, university, and junior and senior students

	Mean	Sd	Median	Iqr	*P*-value
Sex					
M	118.17	18.37	117.5	17	0.0326*
F	112.46	19.13	114	20	
University					
Pavia	120	20.06	118	16	0.0001**
Rome Biomedico Campus	109.77	15.81	110.5	19	
Rome Tor Vergata	111.70	20.81	116	14	
Education					
Junior (I – II years)	112.46	14.31	114	17	0.0253*
Senior (III year)	116.18	26.59	118	24	

*Mann Whitney U test

**Kruskall Wallis test

The differences between categories in the study were all statistically significant.

### Reliability

The final version of the I-NSPIC had a Cronbach’s alpha coefficient of 0.94. Table 5 shows the Cronbach’s alpha values for each of the four subscales. Test–retest analysis in 25 students showed a high degree of reliability for I-NSPIC: ICC = 0.93, *p* < 0.001.

**Table 5:** Reliability: Cronbach’s alpha values for NSPIC and its subscale

	Cronbach’s alpha
NSPIC	0.94
Supportive learning climate	0.95
Instills confidence through caring	0.93
Respectful sharing	0.83
Control vs flexibility	0.69

## Discussion

The aim of the current study was to analyze the validity and reliability of the Italian version of the Nursing Students’ Perception of Instructor Caring (I-NSPIC).

The results of our study show that the factor structure in the original English version cannot be assumed in Italy; in our study we highlight a four-factor structure, rather than five as in the original study and in the Chinese version. The factor that is not confirmed in our study is *Appreciation of life’s meanings*. In addition, some items were loaded onto different factors compared with the original version.

The items related to Appreciation of life’s meanings (“Helps me find personal meaning in my experiences”, “Encourages me to see others’ perspectives about life”, and “Helps me to understand the spiritual dimensions of life”) were loaded in Supportive learning climate. The possible explanation is that the tutorial style and learning methods in Italy direct the phenomenon of care and the idea of human caring through characteristic traits of good care, such as paying attention to others, to feel empathic about the presence and the emotions “Dasein” [47], observing a respectful silence when faced with anguish, in addition to considering the spiritual dimension.

The factor *Supportive learning climate* explains the highest percentage of variability and includes 12 items: six items (“Acknowledge his or her own limitations or mistakes”, “Clearly communicates his or her expectations”, “Serves as a trusted resource for personal problem-solving”, “Offers support during stressful times, Accepts my negative feelings, while helping me to see the positive”, “Allows me to express my true feelings”) are the same as in the original version; three items were loaded from *Appreciation of life’s meanings* and three items from *Instill confidence through caring* (“Instills in me a sense of hopefulness for the future”, “Cares about me as a person”, and “Inspires me to continue the development of knowledge and skills”). We think that in the student’s mind there is no clear subdivision of these three domains (*Supportive learning climate*, *Appreciation of life’s meanings, Instill confidence through caring), m*oreover, the Italian nursing education does not orient the student to understand the appreciation of life’s meanings and the student converts these items into one domain *“Supportive learning climate”*. These differences are mainly determinated by the caring instructor style adopted in the clinical training and its perception among students [5]. In the Italian culture, the tutorial style refers not only to the student’s cognitive processes, but also to the person in its entirety [48, 49].

In fact, as Watson states [50], learning is much more than just acquiring information, facts or data; it implies a meaningful and trusting relationship which is intersubjective. The nature of the relationship, along with the learning form and context, influences the process and demands respect for the individual. In fact, caring interactions between teachers and students reflect the very nature of the professional-client relationship [28]. As Wade and Kasper [27] maintain, nursing students perceptions of their instructors caring can reveal the often unapparent aspects of how nursing students learn to care for their patients. If students perceive an educational climate based on caring, they learn to become professionals.

The factor *Instill confidence through caring* includes seven items: four items (“Shows genuine interest in patients and their care”, “Displays kindness to me and others”, “Makes me feel that I can be successful”, “Helps me envision myself as a professional nurse”) are the same as in the original version; two items (“Respects me as a unique individual” and “Is attentive to me when we communicate”) were loaded from *Respectful sharing;* and one item was loaded from *Supportive learning climate* (“Makes herself/himself available to me”). A possible explanation could be the theoretical assumptions of the andragogy that is well-outlined by Knowless [51] and analyzed in a book in Italian by Franco Angeli, who represents an important reference point for Italian nursing education culture from the 1990s.

The factor, *Respectful Sharing,* includes seven items. In this case only one item is the same as in the original version (12 Does not reveal any of his or her personal side). The remaining items (“Makes me feel like a failure”, “Does not believe in me”, “Makes me nervous in the clinical laboratory”, and “Does not trust my judgment in the clinical laboratory”) were loaded from *Instill confidence through caring* and from *Supportive learning climate* (“Discourages independent problem solving” and “Seems caught up in her/his own priorities rather than responding to my needs”). This result expresses the experience of students who see the importance of instilling respect in the relationship with the clinical tutor. Dynamics probably occur in the relationship between student and clinical tutor, not only at the professional level but the personal one as well, and the student feels it is important to also share personal information with the tutor if this is appropriate and relevant for clinical learning, a factor that has been reported by several authors [15, 20, 21, 24, 26].

The factor *Control* vs *Flexibility* confirms the original version but includes an other item: “Inappropriately discloses personal information about me to others” loaded from *Supportive Learnig climate*. In EFA, this last item had a factor loading smaller than the a priori determined cutoff, but we decided to preserve it in the final questionnaire version because it contributed to construct a factor’s internal reliability*.*

In the student’s mind this behavior of caring instructors is experienced as an element of rigidity rather than as an element of supportive learning. We think that this attitude of instructors caring is perceived by the student as a prejudice that may influence the student’s evaluation process [52].

In other studies as well from other countries [33, 50, 53] with different cultures, which assessed instruments in nursing education, differences have emerged in the factors or the items contained in the model. These results support the explanations in our study.

The inspection of I-NSPIC’s median score revealed that senior students perceived their instructors as caring more than junior students did (median score 118 vs 114 respectively, *p* = 0.0253). Probably senior students perceived their instructors significantly more positively because they have a complete academic experience; therefore they are more confident and aware, unlike junior students who are living in a period of continuous transition. Perhaps the one-to-one relationship in the third year promotes a privileged relationship that improves the interpersonal relationship and facilitates caring and socialization in the profession. In our study, the mean score of the I-NSPIC (113.63) is smaller than the Chinese version C-NSPIC (150.03); therefore the internal consistency of the four factors, except for Control vs *flexibility*, is higher with respect to the C-NSPIC.

### Study limitations

We are aware that our study has some limitations. In this study, we used EFA for exploring the relationships among the variables and we does not have an a priori fixed number of factors; a CFA tests whether a known factor model can predict a set of observed data [54]. Researchers use CFA to verify or confirm hypotheses or theory [55, 56]. In our case, we performed only EFA because we focused our attention on cultural context differences, in fact, our result does not confirm original structure questionnaire. Therefore, our actual work is obtaining more data to implement CFA to confirm or reject the measurement theory get out in this work. Furthermore, in our sample there is no homogenous distribution of males and females (females 80.78%), and thus it is hard to perform subgroup analyses. In addition, the study has a limited geographical representativeness with regard to Italian nursing students (Central and Northern Italy); the participants were limited to nursing students in degree nursing programs. In our opinion, it would be important to know the perception of instructor caring also in diverse several samples such as master’s degree nursing programs and with a sample representing all Italian geographic areas.

## Conclusions

This is the first study to evaluate the psychometric properties of NSPIC in Italy; we have showed that the I-NSPIC has satisfactory reliability and validity. Unlike what is found in the literature [27, 33], our questionnaire presents a four-dimensional structure. We conclude that the I-NSPIC is an effective and valid instrument for assessing the perception of instructor caring in Italian undergraduate nursing students; furthermore it may also prove helpful in promoting the caring ability of nursing students and in increasing the caring interactions in the relationship between instructor and nursing students.

Regarding future research, we feel it is fundamental to use the I-NSPIC questionnaire at the end of each clinical internship that lasts an entire academic year, so as to identify strengths and urgent issues to address regarding the individual tutors in the various internship areas. Moreover, future validation research can expand these findings by testing the psychometric properties of the I-NSPIC on a larger sample from several universities distributed throughout the country, north, centre and sud Italy.

## Additional files

Additional file 1: Questionario “Le percezioni degli studenti infermieri del rapporto con il tutor clinico” (I-NSPIC). (DOCX 27 kb)

Additional file 2: Questionario “Clinical Learning Environment and Supervision plus Nurse Teacher Scale” (CLES-T). (DOC 77 kb)

## Abbreviations

C- NSPIC: Chinese version of the Nursing Students’ Perception of Instructor Caring
CLES-T: Clinical Learning Environment and Supervision plus nurse Teacher scale
EFA: Exploratory Factor Analysis
ICC: Intraclass correlation coefficient
I-NSPIC: Italian version of the Nursing Students’ Perception of Instructor Caring
NSPIC: Nursing Students’ Perception of Instructor Caring
