# Supplementary material for: Italian version of Nursing Students’ Perception of Instructor Caring (I-NSPIC): assessment of reliability and validity
Source: BMC Med Educ. 2017 Nov 17;17:218. doi: 10.1186/s12909-017-1032-y (PMC5693487; doi:10.1186/s12909-017-1032-y)
Supplement: Supplementary file 1 — Questionario “Le percezioni degli studenti infermieri del rapporto con il tutor clinico” (I-NSPIC). (DOCX 27 kb) [file 12909_2017_1032_MOESM1_ESM.docx]

**Additional file 1**

**Socio demographic variables and questionnaire about Perception of Instructor Caring**

**QUESTIONARIO**

**"LE PERCEZIONI DEGLI STUDENTI INFERMIERI DEL RAPPORTO CON IL TUTOR CLINICO"**

Gentile Studentessa, Gentile Studente

Le chiediamo di compilare il questionario seguendo le istruzioni e facendo riferimento all'anno di corso frequentato nell'anno accademico 2014/2015.

**DATI DEL COMPILATORE**

**Età: ...............**

**Sesso:** o **maschile** o **femminile Anno di nascita:** ...........................

**Anno di corso:** o I **anno** o Il **anno** o **lII anno Regione di provenienza:** ...............................................

**Università di provenienza:** ..........................................

**Sede di corso:**…………………………………………………

*Versione italiana del "Nursing Student Perception of instructor caring lnstrument" {NSPIC) (Wàde and Kasper, J Nurs Edue 2006)*

**"LE PERCEZIONI DEGLI STUDENTI INFERMIERI DEL RAPPORTO CON IL TUTOR CLINICO"**

Istruzioni per la compilazione: legga i seguenti item pensando al suo tutor clinico attuale.

Faccia un segno sul numero che meglio rappresenta la sua opinione.

| Il mio tutor clinico: | | | Forte disaccordo | Moderato disaccordo | Leggero disaccordo | Leggero accordo | Moderato accordo | Forte accordo |
| --- | --- | --- | --- | --- | --- | --- | --- | --- |
| 1. Mostra un autentico interesse per i pazienti e per la loro assistenza | |  | 1 | 2 | 3 | 4 | 5 | 6 |
| 2. E' gentile con me e con gli altri |  |  | 1 | 2 | 3 | 4 | 5 | 6 |
| 3. Mi infonde speranza nel futuro | | | 1 | 2 | 3 | 4 | 5 | 6 |
| 4. Mi fa sentire che ho le capacità per avere successo | | | 1 | 2 | 3 | 4 | 5 | 6 |
| 5. Mi aiuta a immaginarmi come infermiere | | | 1 | 2 | 3 | 4 | 5 | 6 |
| 6. Mi fa sentire un fallimento | | | 1 | 2 | 3 | 4 | 5 | 6 |
| 7. Non crede in me | | | 1 | 2 | 3 | 4 | 5 | 6 |
| 8. Si prende cura di me come persona | | | 1 | 2 | 3 | 4 | 5 | 6 |
| 9. Mi rispetta come persona | | | 1 | 2 | 3 | 4 | 5 | 6 |
| 10. Quando comunichiamo mi presta attenzione | | | 1 | 2 | 3 | 4 | 5 | 6 |
| 11. Divulga inappropriatamente informazioni che riguardano il mio lato personale | | | 1 | 2 | 3 | 4 | 5 | 6 |
| 12. Non manifesta niente su di sé | | | 1 | 2 | 3 | 4 | 5 | 6 |
| 13. Riconosce i suoi limiti o errori | | | 1 | 2 | 3 | 4 | 5 | 6 |
| 14. Si rende disponibile nei miei confronti | | | 1 | 2 | 3 | 4 | 5 | 6 |
| 15. Comunica in modo chiaro le sue aspettative | | | 1 | 2 | 3 | 4 | 5 | 6 |
| 16. E' un riferimento affidabile per affrontare problemi personali | | | 1 | 2 | 3 | 4 | 5 | 6 |
| 17. Offre il suo supporto nei momenti di stress | | | 1 | 2 | 3 | 4 | 5 | 6 |
| 18. Accoglie i miei sentimenti negativi e mi aiuta a *vedere* il lato positivo delle cose | | | 1 | 2 | 3 | 4 | 5 | 6 |
| 19. Mi permette di esprimere i miei sentimenti | | | 1 | 2 | 3 | 4 | 5 | 6 |
| 20. Scoraggia la risoluzione autonoma dei problemi | | | 1 | 2 | 3 | 4 | 5 | 6 |
| 21. Mi ispira a continuare nel mio sviluppo di conoscenze e di abilità | | | 1 | 2 | 3 | 4 | 5 | 6 |
| 22. In tirocinio clinico mi rende nervoso | | | 1 | 2 | 3 | 4 | 5 | 6 |
| 23. In tirocinio clinico non si fida della mia capacità di giudizio | | | 1 | 2 | 3 | 4 | 5 | 6 |
| 24. Sembra preso più dalle sue priorità, che dai miei bisogni | | | 1 | 2 | 3 | 4 | 5 | 6 |
| 25. Mi esige tale disponibilità di tempo da interferire con le mie necessità fondamentali | | | 1 | 2 | 3 | 4 | 5 | 6 |
|  | | |  |  |  |  |  |  |
| 26. Si concentra sul completamento dei compiti assistenziali piuttosto che sui bisogni del paziente | | | 1 | 2 | 3 | 4 | 5 | 6 |
| 27. Mi aiuta a trovare un significato personale alle mie esperienze | | | 1 | 2 | 3 | 4 | 5 | 6 |
| 28. Mi incoraggia a vedere la *vita* anche dal punto di vista degli altri | | | 1 | 2 | 3 | 4 | 5 | 6 |
| 29. Mi aiuta a comprendere le dimensioni spirituali della vita | | | 1 | 2 | 3 | 4 | 5 | 6 |
| 30. E' rigido quando è messo di fronte a situazioni o avvenimenti inattesi | | | 1 | 2 | 3 | 4 | 5 | 6 |
| 31. Utilizza i voti per mantenere il controllo sugli studenti | | | 1 | 2 | 3 | 4 | 5 | 6 |

Il questionario è terminato. Grazie della collaborazione.

*Versione italiana del "Nursing Student Perception of instructor caring lnstrument" (NSPIC)*

*(Wade and Kasper, J Nurs Educ 2006}*
