# Supplementary material for: Italian version of Nursing Students’ Perception of Instructor Caring (I-NSPIC): assessment of reliability and validity
Source: BMC Med Educ. 2017 Nov 17;17:218. doi: 10.1186/s12909-017-1032-y (PMC5693487; doi:10.1186/s12909-017-1032-y)
Supplement: Supplementary file 2 — Questionario “Clinical Learning Environment and Supervision plus Nurse Teacher Scale” (CLES-T). (DOC 77 kb) [file 12909_2017_1032_MOESM2_ESM.doc]

**Additional file 2**

**Social variables and questionnaire about clinical learning environment and supervisory relationship between tutor and student**

**CLINICAL LEARNING ENVIRONMENT AND SUPERVISION PLUS NURSE TEACHER SCALE (*CLES - T*)**

Data di compilazione:…………………

Indicare la propria risposta o scrivere quanto richiesto negli spazi predisposti

**Dati del Compilatore**

Esperienze lavorative: □ sì, pregresse □ sì, in corso □ no

In possesso di titolo di OSS (operatore socio sanitario): □ si □ no

Nazionalità: ………………………….

I seguenti items si riferiscono all’**unità operativa** nella quale è stata **appena completata** l’esperienza di tirocinio.

| Unità operativa/Servizio sede di tirocinio:…………………………………………. |
| --- |
| Periodo di tirocinio:……………………………………….. |
| L’**unità operativa** è già stata frequentata da te in precedenti tirocini?  □ Sì □ No |
| Degenza media del paziente nell’unità operativa:  □ < 1 settimana  □ Da 1 a 2 settimane  □ Da 2 a 3 settimane  □ Da 3 a 4 settimane  □ Da 1 a 2 mesi  □ Oltre 2 mesi |
| Durata del tirocinio in settimane: …..………………… |
| Quanti incontri ci sono stati con il tutor universitario durante il tirocinio?....................................... |
| Durante il tirocinio sono subentrati eventi che hanno interrotto la continuità dell’esperienza clinica o del tutorato? (ad esempio malattia del tutor clinico, tutor universitario, studente, motivi personali …)  □ Sì □ No |
| Sono stati utilizzati strumenti di comunicazione elettronica con il tutor universitario durante il tirocinio?  □ No □ Sì, specificare il numero …... |
| Esito dell’eventuale valutazione intermedia di tirocinio:  □ Positivo □ Negativo □ Non si è svolta |
| È già avvenuta la valutazione finale di questo tirocinio?  □ Sì □ No |

**CLINICAL LEARNING ENVIRONMENT AND SUPERVISION PLUS NURSE TEACHER SCALE (*CLES - T*)**

Questa scala misura alcuni aspetti relativi all’ambiente di apprendimento durante lo svolgimento del tirocinio clinico.

Legenda:

Indicare il grado di accordo/disaccordo relativo ai seguenti item.

Scala di valutazione:

1= forte disaccordo; 2=disaccordo; 3=né accordo né disaccordo; 4=accordo; 5=forte accordo

Indicare il numero corrispondente alla propria risposta o scrivere quanto richiesto negli spazi predisposti

| **Clima di apprendimento nel reparto** | | | | | |
| --- | --- | --- | --- | --- | --- |
| 1. Tutta l’équipe si è dimostrata disponibile nei miei confronti. | 1 | 2 | 3 | 4 | 5 |
| 2. Durante i momenti di discussione sui pazienti (es consegne, discussione dei casi) mi sono sentito a mio agio nel prendere parte alla discussione. | 1 | 2 | 3 | 4 | 5 |
| 3. Mi recavo volentieri in reparto per iniziare il turno di tirocinio. | 1 | 2 | 3 | 4 | 5 |
| 4. Nel reparto c’era un clima positivo. | 1 | 2 | 3 | 4 | 5 |
| 5. Tutta l’équipe è stata partecipe del mio apprendimento clinico. | 1 | 2 | 3 | 4 | 5 |
| 6. L’équipe si rivolgeva a me usando il mio nome. | 1 | 2 | 3 | 4 | 5 |
| 7. Nel reparto ci sono state sufficienti e significative occasioni di apprendimento. | 1 | 2 | 3 | 4 | 5 |
| 8. Le occasioni di apprendimento sono state multi-dimensionali, ovvero varie in termini di contenuto. | 1 | 2 | 3 | 4 | 5 |
| 9. Il reparto può essere considerato un buon ambiente di apprendimento. | 1 | 2 | 3 | 4 | 5 |
| **Stile di leadership del Coordinatore Infermieristico** | | | | | |
| 10. Il Coordinatore considerava l’équipe del suo reparto una risorsa determinante per la qualità dell’assistenza. | 1 | 2 | 3 | 4 | 5 |
| 11. Il Coordinatore era un membro del team (“uno di loro”). | 1 | 2 | 3 | 4 | 5 |
| 12. I feedback del Coordinatore erano considerati agevolmente dall’équipe come occasioni di apprendimento. | 1 | 2 | 3 | 4 | 5 |
| 13. I contributi dei singoli membri dell’équipe erano apprezzati. | 1 | 2 | 3 | 4 | 5 |
| **Erogazione dell’assistenza infermieristica nel reparto** | | | | | |
| 14. Il modello di assistenza infermieristica in reparto era ben definito. | 1 | 2 | 3 | 4 | 5 |
| 15. I pazienti ricevevano assistenza infermieristica personalizzata. | 1 | 2 | 3 | 4 | 5 |
| 16. Non c’erano problemi nei flussi di informazioni correlati all’assistenza dei pazienti. | 1 | 2 | 3 | 4 | 5 |
| 17. La documentazione infermieristica (es. diario infermieristico) era di chiaro utilizzo. | 1 | 2 | 3 | 4 | 5 |

| **La relazione di tutorato con il personale di reparto** | | | | | | | | | | | |
| --- | --- | --- | --- | --- | --- | --- | --- | --- | --- | --- | --- |
| *In quest’area il concetto di tutorato clinico si riferisce al guidare, supportare e valutare gli studenti infermieri da parte del personale di reparto. Il tutorato clinico può essere di tipo individuale o inteso come funzione diffusa di tutta l’équipe di reparto. Il tutor clinico è l’infermiere esperto che si occupa del tutorato dello studente nella specifica realtà clinica*. | | | | | | | | | | | |
| 18. Il mio tutor clinico ha dimostrato atteggiamenti positivi verso il tutorato. | | 1 | | 2 | | 3 | | 4 | | 5 | |
| 19. Ho sentito di aver ricevuto un tutorato personalizzato. | | 1 | | 2 | | 3 | | 4 | | 5 | |
| 20. Ho ricevuto feedback continui dal mio tutor clinico. | | 1 | | 2 | | 3 | | 4 | | 5 | |
| 21. Complessivamente sono soddisfatto del tutorato ricevuto. | | 1 | | 2 | | 3 | | 4 | | 5 | |
| 22. Il tutorato clinico è stato fondato su una relazione equa e ha promosso in mio apprendimento. | | 1 | | 2 | | 3 | | 4 | | 5 | |
| 23. Nella relazione di tutorato c’è stata un’interazione reciproca. | | 1 | | 2 | | 3 | | 4 | | 5 | |
| 24. Nella relazione tra tutor clinico e studente hanno prevalso reciproco rispetto e riconoscimento. | | 1 | | 2 | | 3 | | 4 | | 5 | |
| 25. La relazione di tutorato è stata caratterizzata da un senso di fiducia. | | 1 | | 2 | | 3 | | 4 | | 5 | |
| **Ruolo del tutor universitario** | | | | | | | | | | | |
| *Per tutor universitario si intende un tutor assegnato a svolgere il proprio ruolo presso l’università.* | | | | | | | | | | | |
| 26. Secondo me il tutor universitario è stato capace di integrare la conoscenza teorica con la pratica infermieristica quotidiana. | 1 | | 2 | | 3 | | 4 | | 5 | |  |
| 27. Il tutor universitario è stato capace di dare concretezza agli obiettivi d’apprendimento di questo tirocinio | 1 | | 2 | | 3 | | 4 | | 5 | |  |
| 28. Il tutor universitario mi ha aiutato a ridurre il gap teoria-pratica. | 1 | | 2 | | 3 | | 4 | | 5 | |  |
| 29. Il tutor universitario è considerato parte dell’équipe infermieristica di reparto. | 1 | | 2 | | 3 | | 4 | | 5 | |  |
| 30. Il tutor universitario è stato in grado di condividere la sua competenza pedagogica con l’équipe infermieristica del reparto. | 1 | | 2 | | 3 | | 4 | | 5 | |  |
| 31. Il tutor universitario e l’équipe del reparto hanno lavorato insieme per favorire il mio apprendimento. | 1 | | 2 | | 3 | | 4 | | 5 | |  |
| 32. Gli incontri periodici fra me, il tutor clinico e il tutor universitario sono state esperienze gradevoli. | 1 | | 2 | | 3 | | 4 | | 5 | |  |
| 33. Il clima che si è creato durante gli incontri è stato partecipativo. | 1 | | 2 | | 3 | | 4 | | 5 | |  |
| 34. Gli incontri erano focalizzati sui miei bisogni di apprendimento. | 1 | | 2 | | 3 | | 4 | | 5 | |  |

*Ti chiediamo, infine, di compilare la griglia seguente:*

| **Modelli di ruolo nella formazione infermieristica** |
| --- |
| 35. Ruolo istituzionale del tutor clinico:  □ Infermiere  □ Infermiere specialista  □ Infermiere di supporto al Coordinatore infermieristico  □ Coordinatore infermieristico  □ Altro (specificare):………………….. |
| 36. Modalità di tutorato clinico (indicare un’unica opzione)  □ Non avevo un tutor  □ È’ stato nominato un tutor personale (rapporto di tutorato 1:1 o 1:2)  □ Il tutor è cambiato in base al turno  □ Lo stesso tutor ha seguito più studenti (rapporto di tutorato 1:3 o maggiore)  □ Altre modalità di tutorato (specificare):……………………………. |
| 37. Ci sono stati incontri di tutorato individuale con il tutor clinico (senza la presenza del tutor universitario)?  □ No  □ Una o due volte durante il tirocinio  □ Meno di una volta a settimana  □ Circa una volta a settimana  □ Più spesso |
| 38. Nella tua esperienza, qual è stata la persona che più delle altre ti ha aiutato a comprendere i concetti chiave della disciplina e della pratica infermieristica?  □ Il tutor clinico  □ Il tutor universitario  □ Entrambi |
| 39. All’inizio di questo tirocinio  □ Non ero motivato  □ Ero motivato  □ Ero altamente motivato |
| 40. Quanto sono soddisfatto del tirocinio appena concluso?  □ Molto insoddisfatto  □ Piuttosto insoddisfatto  □ Né soddisfatto né insoddisfatto  □ Piuttosto soddisfatto  □ Molto soddisfatto |
